# Editorial: Applications of medicine in treating pulmonary fibrosis

**DOI:** 10.3389/fphar.2023.1212681

**Published:** 2023-07-04

**Authors:** Wenjun Li, Xin Shi, Changjun Lv, Haibo Hu, Isaac Kirubakaran Sundar, Song Qin

**Affiliations:** ^1^ Yantai Institute of Coastal Zone Research, Chinese Academy of Sciences, Yantai, China; ^2^ Shandong Technology and Business University, Yantai, China; ^3^ Binzhou Medical University Hospital, Binzhou Medical University, Binzhou, China; ^4^ Qingdao Hospital of Traditional Chinese Medicine (Qingdao Hiser Hospital), Qingdao, China; ^5^ Department of Internal Medicine, Division of Pulmonary Critical Care and Sleep Medicine, University of Kansas Medical Center, Kansas City, KS, United States

**Keywords:** pulmonary fibrosis, traditional medicine, synthetic drugs, biological drugs, clinical trials

Pulmonary fibrosis (PF) is a pathology characterized by an inflammatory response and abnormal deposition of extracellular matrix, which manifests as progressive and irreversible damage to lung tissue structures, ultimately leading to organ dysfunction, respiratory failure, and death (Meyker, 2017). The inflammatory response, oxidative stress, and epithelial-to-mesenchymal transition are important factors that predispose to the development and progression of pulmonary fibrosis (Moss et al., 2022). Therefore, drugs that target these pathological alterations will facilitate the development of PF therapy. Glucocorticoids and antioxidants are used in the early treatment of pulmonary fibrosis, which could reduce the inflammatory response and oxidative stress in the lungs and can prevent the pathological changes of tissue fibrosis (Spagnolo et al., 2021). However, these drugs only slow down the decline in lung function and do not reverse the histopathological changes in the lung. Currently, lung transplantation is considered to be the only effective treatment, but the complex etiology and limited availability of donor organs make it difficult to achieve effective treatment. There is lack of new therapies for the treatment of pulmonary fibrosis and to elucidate the underlying mechanisms and potential targets for the pathogenesis of pulmonary fibrosis.

Pirfenidone is an approved drug with efficacy in PF, which reduces organ fibrosis by inhibiting collagen synthesis, decreasing inflammatory mediators, and reducing oxidative stress, and is widely used in clinical practice (Raghu, 2017). However, the adverse effects associated with pirfenidone vary considerably between individuals, and the dose is not fixed. A study by Li et al. evaluated the efficacy and safety of different plasma concentrations of pirfenidone in patients with pulmonary fibrosis, and found that high doses of pirfenidone (1800 mg/d) reduced the risk of exacerbation in patients with PF, while provoking side effects in the gastrointestinal system and the skin. However, the presence of these side effects did not discontinue treatment in patients with PF, as the high concentration of pirfenidone is beneficial for the retardation of the patient’s condition (Li et al.).

Traditional Chinese medicine (TCM) is an important part of clinical practice and can be used in combination with each other to achieve a reduction in toxicity and an increase in effectiveness. The exploration and application of TCM in the treatment of PF are gradually increasing. Numerous researchers have found that the progression of PF is closely related to gut microbiota changes, which also suggests that regulating the gut microbiota could provide a new strategy for the treatment of PF. For example, in this Research Topic, Hu et al. evaluated Bu-Fei-Huo-Xue capsules significantly improved collagen deposition, reduced levels of inflammatory factors in the lungs, and inhibited oxidative stress in mice with PF. It is interesting that Bu-Fei-Huo-Xue capsules also affected the diversity and relative abundance of the intestinal microbiota (Hu et al.), suggesting that the mechanism of Bu-Fei-Huo-Xue capsules on PF may be related to the regulation of gut microbiota. Based on a combination of net pharmacology and animal models, Zuo et al. revealed that Yiqi Huayu decoction was effective in reducing lung tissue damage, improving lung function, reducing inflammatory responses, and decreasing aging-related secretory phenotypes, including interleukin (IL)-1L and transforming growth factor-β1 (TGF-β1) in rats with PF. Similarly, Xiong et al. determined arctiin as an active component from *Arctium lappa* L. to exert therapeutic activity against PF by network pharmacology and prepared arctigenin nanoparticles. The result showed that arctiin nanoparticles exhibited good anti-aging properties *in vivo* and could attenuate AEC II senescence and PF via inhibiting the p38/p53/p21 pathway (Xiong et al.). The use of new technologies combined with conventional animal models to systematically and comprehensively elucidate the relationship between PF and disease targets, and between targets and drugs, will hopefully overcome the challenge of therapeutic strategies lagging behind medical advances and clinical needs.

The pursuit of an effective therapeutic target is the most important approach in anti-fibrotic drug discovery. Researchers have described several targets in this Research Topic. Long et al. reported that specific deletion of FGFR1 was able to protect lung tissue from hyperoxia injury, which may be attributed to the regulation of FGFR1 on both common and aerosol capillary endothelial cells, improving vascular and alveolar generation as well as respiratory indexes. In addition, the target-based discovery of small molecule drugs is an important research direction for the treatment of PF. Solopov et al. demonstrated that TAS-116, an HSP-90 inhibitor, could serve as an effective antidote against lung injury induced by hydrochloric acid, and it was able to reduce the overexpression of NLRP3 inflammasome and inhibit the activation of profibrotic pathways during the peak period of lung injury, thereby exerting lung protective activity. The cAMP and cGMP are intracellular second messengers that play important roles in multiple physiological functions. Yang et al. concluded that phosphodiesterase is involved in the metabolism of nucleotides, and its inhibitors can increase the concentration of intracellular cyclic nucleotides, thereby regulating cAMP and cGMP for their anti-fibrotic effects in the lungs. Moreover, Chen et al. reported that umbilical cord MSCs-hepatocyte growth factor would be a promising therapeutic option for PF, as over-expression of hepatocyte growth factor could enhance the anti-fibrotic effects of umbilical cord MSCs by interacting with IL-17-producing cells in lung tissue. A number of studies have revealed that senescent phenotypes such as aberrant telomere shortening seen in alveolar type II epithelial cells promote the development of PF (Duckworth et al., 2021). Zhang et al. show that Telodin, a newly discovered small molecule peptide inhibitor of telomere dysfunction, could reduce telomere shortening, expand the mouse AEC II stem cell population, and prevent chronic stress-induced premature lung senescence and fibrosis, which will promisingly be a new strategy to terminate pulmonary fibrosis.

We collectively identified a range of attractive therapeutic agents for PF, including pirfenidone, traditional Chinese medicine, and targeted novel small molecule inhibitors (Figure 1). Many studies have also presented complex gene-target-chemical components and molecular mechanisms underlying the development of PF. All these findings will provide lead structures for the development of anti-fibrotic drugs and further define their efficacy and pharmacokinetics *in vivo*. Further research on which specific anti-fibrotic drugs benefit patients with PF will help medical personnel to develop a more rational individualized protocol for the diagnosis and treatment.

**FIGURE 1:**
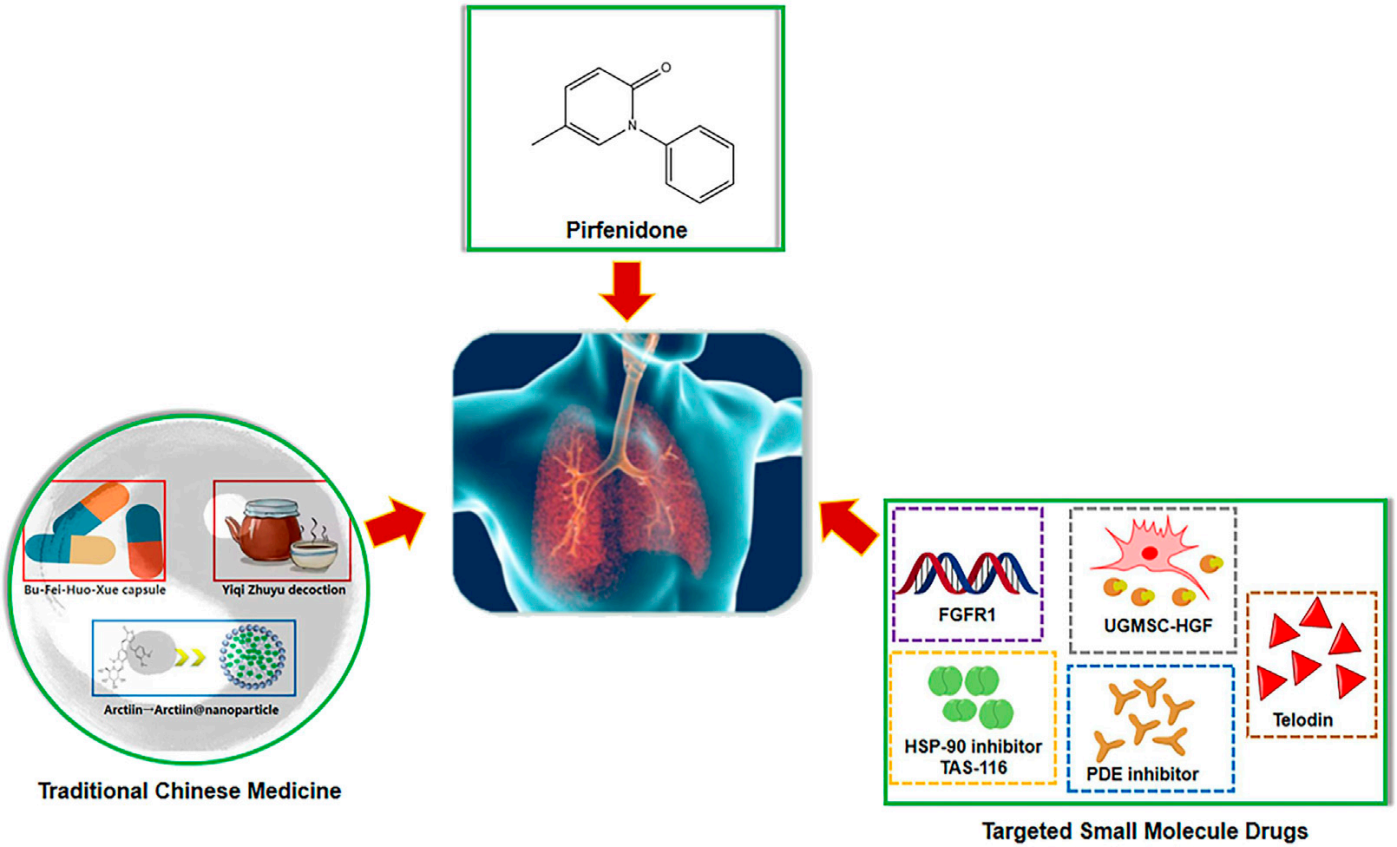
The drugs used to treat pulmonary fibrosis in this Research Topic include pirfenidone, traditional Chinese medicine and other novel small molecule drugs that target pro-fibrotic phenotypes.

## References

[B1] Duckworth A. Gibbons M. A. Allen R. J. Almond H. Beaumont R. N. Wood A. R. (2021). Telomere length and risk of idiopathic pulmonary fibrosis and chronic obstructive pulmonary disease: A mendelian randomisation study. Lancet. Respir. Med. 9 (3), 285–294. 10.1016/S2213-2600(20)30364-7 33197388

[B2] Meyer K. C. (2017). Pulmonary fibrosis, part I: Epidemiology, pathogenesis, and diagnosis. Expert Rev. Respir. Med. 11 (5), 343–359. 10.1080/17476348.2017.1312346 28345383

[B3] Moss B. J. Ryter S. W. Rosas I. O. (2022). Pathogenic mechanisms underlying idiopathic pulmonary fibrosis. Annu. Rev. pathology 17, 515–546. 10.1146/annurev-pathol-042320-030240 34813355

[B4] Raghu G. (2017). Pharmacotherapy for idiopathic pulmonary fibrosis: Current landscape and future potential. Eur. Respir. Rev. official J. Eur. Respir. Soc. 26 (145), 170071. 10.1183/16000617.0071-2017 PMC9488742 28954769

[B5] Spagnolo P. Kropski J. A. Jones M. G. Lee J. S. Rossi G. Karampitsakos T. (2021). Idiopathic pulmonary fibrosis: Disease mechanisms and drug development. Pharmacol. Ther. 222, 107798. 10.1016/j.pharmthera.2020.107798 33359599 PMC8142468

